# Carbonate of Soda, in Whooping Cough

**Published:** 1878-05

**Authors:** 


Carbolate of Soda in Whooping-cough.—M. Pernot (*Lyon Medicale*, Sept. 23, 1877) considers that he has discovered a specific for this troublesome affection in "phènate de soude," and gives details of cases in which, after other means had completely failed, he was able, by the use of it, to effect a complete cure in from ten to fourteen days. He places about 40 grammes of the crude salt in a porcelain capsule, and heats it over a spirit lamp so as to disengage carbolic vapours, the child being kept in the vapour a short time at first, and a longer time as he becomes more accustomed to it. In the most rebellious cases he has not required to use the treatment more than three times a day, and in most cases it has only been necessary to use it night and morning. He discusses the mode of preparation of carbolic acid and its salts, and ascribes the curative properties of the phenate of soda to the tarry compounds which it contains. "My observations," he says, "are now numerous; they, for the most part, resemble each other, and, speaking generally, we may sum up the results in the following words: 1st. There is a notable diminution in the number of 'kinks' after two to ten days' treatment. 2d. The respiration is less painful, less anxious. 3d. The 'kinks are of shhrter duration. 4th. There is less vomiting, possibly because the 'kinks' are shorter. 5th. Finally, the most stubborn cases, if I may so express myself, cease to advance from the commencement of the treatment, then diminish in intensity, little by little, and afterwards more rapidly."—*Glasgow Med. Journal*, Jan. 1878.

